# Thiol-functionalized magnetite/graphene oxide hybrid as a reusable adsorbent for Hg^2+^ removal

**DOI:** 10.1186/1556-276X-8-486

**Published:** 2013-11-19

**Authors:** Jian Bao, You Fu, Zhihao Bao

**Affiliations:** 1 Jiangsu Provincial Academy of Environmental Science, 241 Fenghuang West Street, Nanjing, Jiangsu 210036, China; 2 Shanghai Key Laboratory of Special Artificial Microstructure Materials and Technology, Tongji University, 1239 Siping Road, Shanghai 200092, China

**Keywords:** Mercury ion, Magnetite, Adsorption capacity, Graphene oxide, Hybrid

## Abstract

A thiol-functionalized magnetite/graphene oxide (MGO) hybrid as an adsorbent of Hg^2+^ was successfully synthesized by a two-step reaction. It exhibited a higher adsorption capacity compared to the bare graphene oxide and MGO due to the combined adsorption of thiol groups and magnetite nanocrystals. Its capacity reached 289.9 mg g^-1^ in a solution with an initial Hg^2+^ concentration of 100 mg l^-1^. After being exchanged with H^+^, the adsorbent could be reused. The adsorption of Hg^2+^ by the thiol-functionalized MGO fits well with the Freundlich isotherm model and followed pseudo-second-order kinetics.

## Background

Due to the development and expansion of industry, pollution of heavy metals in water supplies increases in the recent years. The pollution is seriously threatening the ecological systems as well as human health. Among them, mercury is one of the most hazardous elements due to its toxicological and biogeochemical behavior [1,2]. A lot of adsorbents have been employed to extract Hg^2+^ from the industrial wastewaters. For example, thiol-functionalized adsorbents exhibited a specific binding capability toward highly toxic heavy metal ions including Hg^2+^ due to the existence of the thiol groups [3-6]. While for iron oxides, their adsorption mechanism was attributed to the complexation of Hg^2+^ and surface hydroxyl group at the iron oxide/water interface [7-9]. Iron oxide nanocrystals can further enhance the adsorption capacities because of their high specific surface area [6,10]. Another advantage of using iron oxide-based adsorbents is that they can be easily extracted from wastewater by applying an external magnetic force. However, few research works have reported on adsorbents with both adsorption effects. The emergence of graphene oxide makes such combination possible due to its abundant functional moieties (hydroxyl and carboxyl groups) [11,12], which enable possible metal oxide deposition and functional organic group grafting on its surface [13-15]. In this work, we deposited Fe_3_O_4_ nanoparticles on graphene oxide and then grafted thiol groups on the Fe_3_O_4_/graphene oxide (MGO). The thiol-functionalized MGO exhibited relatively high Hg^2+^ adsorption capacity. The adsorbent could be separated from the water solutions easily and reused after it was exchanged with H^+^.

## Methods

### Chemicals and materials

Natural graphite (500 mesh), 98 wt.% H_2_SO_4_, 5 wt.% HCl aqueous solution, 30 wt.% H_2_O_2_ aqueous solution, acetone, and Na_2_CO_3_ were purchased from Sinopharm Chemical Reagent Co., Ltd. (Shanghai, China). 1-Methyl-2-pyrrolidone (NMP), ferric acetylacetonate (Fe(acac)_3_), potassium permanganate (KMnO_4_), NaHCO_3_, 1-ethy-3-(3-dimethyllaminopropyl) carvodiimide hydrochloride (EDC), and 2-mercaptoethylamine (MEA) were purchased from Aladdin Reagent Company (Shanghai, China). Other reagents used were of analytical grades without further purification. Deionized water was used in all the processes of aqueous solution preparations.

### Preparation of MGO

Graphene oxide (GO, 100 mg) was dispersed in 30 ml of NMP by ultrasonication at room temperature, and the mixture was heated to 190°C under an argon atmosphere. Fe(acac)_3_ (1.413 g, 4 mmol) was dissolved in 20 ml of NMP and added dropwise in about 1 h to the GO/NMP solution under vigorous stirring. The stirring was continued for another 4 h after the dropping was finished. After being cooled to room temperature, the mixture was washed three times using acetone and water alternatively. The precipitate was collected by magnetic separation and was then dispersed in water by ultrasonication. The resulting black powder was collected by freeze-drying.

### Synthesis of thiol-functionalized MGO

MGO (10 mg) was dispersed in 10 ml of deionized water by ultrasonication in an ice bath. EDC of 50 ml and a Na_2_CO_3_-NaHCO_3_ (1:9) buffer solution were added to adjust the pH of the system to approximately 9. After carboxyl groups on MGO were activated in 1 h, a solution containing 100 mg of MEA was added dropwise to the system. With the protection of argon, the reaction lasted for 24 h. The precipitate was collected by magnetic separation and was then dispersed in water by ultrasonication. The resulting black powder was collected by freeze-drying.

### Adsorption experiment

The effects of the initial concentration of Hg^2+^ and adsorption time on the final adsorption capacity were tested to obtain the saturated adsorption capacity and dynamic adsorption curve. Thiol-functionalized MGO powder was added to 25 ml of water solution with different concentrations of Hg^2+^. NaOH was used to adjust the pH of the solution. While the temperature was kept stable by using a water bath, the samples were placed on a standard rocker and oscillated for given hours. The supernate was collected by magnetic separation for reproducibility test. After washing with diluted HCl (0.25 N), the thiol-functionalized MGO was re-immersed in the solution with an initial Hg^2+^ concentration of 100 mg l^-1^ and oscillated for 48 h.

### Characterization

The X-ray diffraction (XRD) pattern was taken on a D/MAX-RB diffractometer using Cu Kα radiation. Investigation of the microstructure was performed by transmission electron microscopy (TEM, JEOL JEM-2010 F, JEOL Ltd., Akishima, Tokyo, Japan). Water bath sonication was performed with a JYD 1800 L sonicator (100 to 2,000 W, ZhiXin Instrument Co., Ltd, Shanghai, China). Hg^2+^ concentration was determined by using a DMA-80 direct mercury analyzer (Milestone S.r.l., Sorisole, Italy).

## Results and discussion

**Figure 1:**
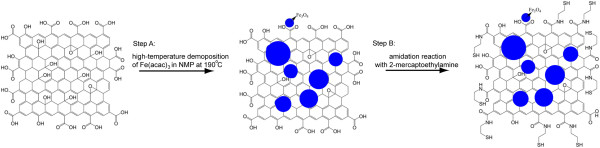
Schematic of synthesis of thiol-functionalized MGO from graphene oxide.

**Figure 2:**
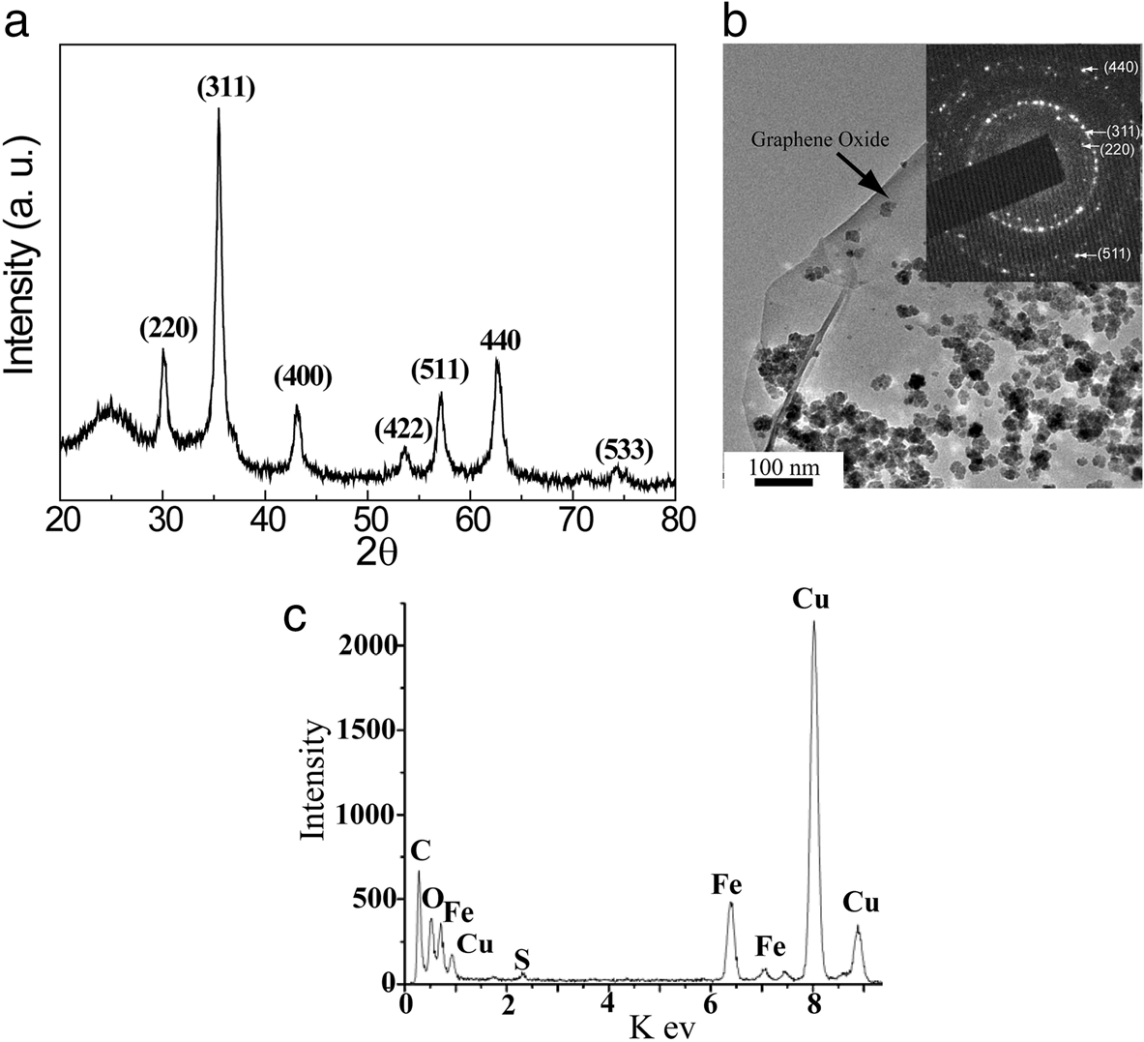
**XRD pattern, TEM image, and EDAX analysis. (a)** XRD pattern of MGO, **(b)** TEM image of MGO (inset, the electron diffraction pattern of MGO), and **(c)** EDAX analysis of thiol-functionalized MGO.

**Figure 3:**
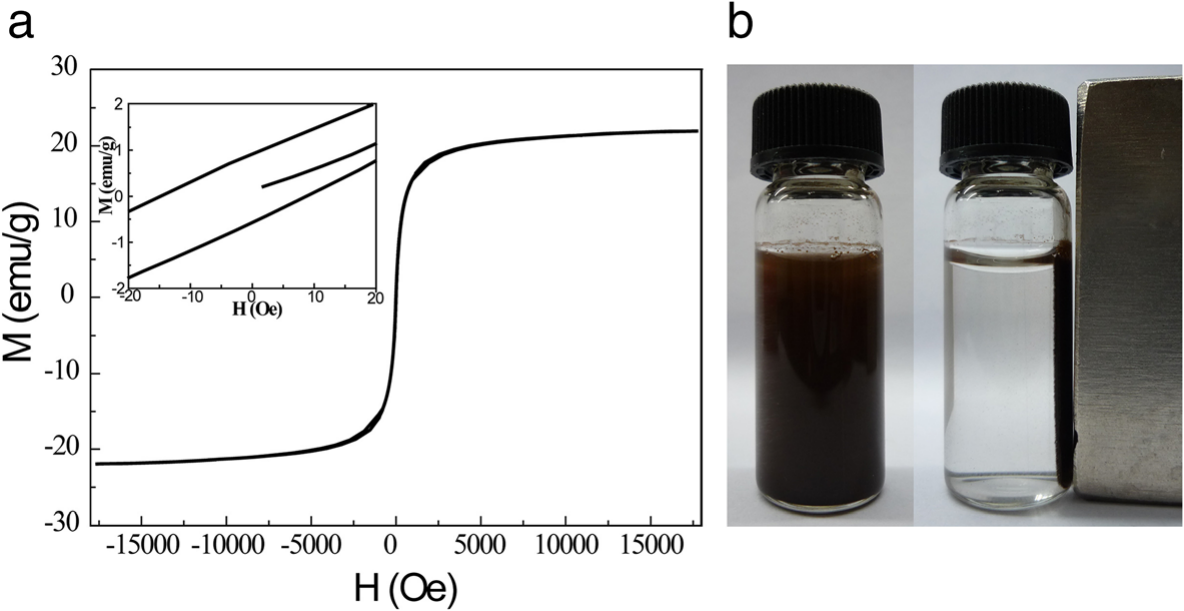
**Hysteresis loop and extraction of the thiol-functionalized MGO. (a)** Hysteresis curve of thiol-functionalized MGO (inset, close view of hysteresis loops) and **(b)** the water solution dispersed with thiol-functionalized MGO and magnetic separation.

GO was prepared from natural graphite using modified Hummer's method [16,17]. Fe_3_O_4_ nanoparticles were deposited on graphene oxide by decomposition of Fe(acac)_3_ in NMP solution (Figure 1, step A) at 190°C [18]. Figure 2a shows the XRD pattern of the product. The peaks at 30.2°, 35.5°, 43.1°, 53.5°, 57.0°, 62.4° in the pattern could be ascribed to diffraction of (220), (311), (400), (422), (511), and (440) crystal planes of Fe_3_O_4_ (magnetite, JCPDS no. 75–0033). Based on the Scherrer analysis of the pattern, the crystallite size of Fe_3_O_4_ was estimated to be 13.0 nm. The appearance of the magnetite phase was consistent with the electron diffraction pattern (inset in Figure 2b). The TEM image (Figure 2b) of the product showed that GO was decorated with magnetite aggregates with a size of several tens of nanometers. In the synthesis process, carbon monoxide was generated at a relatively high temperature and partially reduced Fe^3+^ to Fe^2+^. Then, the magnetite nanocrystals nucleated and grew at the oxygen-containing defects sites such as carboxyl, hydroxyl, and epoxy groups [14]. Finally, MGO was obtained. Thiol functional groups were grafted on the MGO by the reaction between MEA and carboxyl groups on GO activated by EDC (Figure 1, step B). Energy-dispersive X-ray spectroscopy (EDAX) analysis (Figure 2c) indicated the appearance of the sulfur element, indicating that the thiol groups were successfully grafted on MGO. Thus, the thiol-functionalized MGO was obtained after the reaction. The magnetic properties of the thiol-functionalized MGO were investigated using a superconducting quantum interference device (SQUID) magnetometer. Figure 3 shows the hysteresis loop of the thiol-functionalized MGO hybrids at room temperature (300 K). The saturation magnetization was 22.0 emu g^-1^, which was much smaller than 92.0 emu g^-1^, the saturation magnetization of bulk Fe_3_O_4_[19]. The reduction in the value of saturation magnetization could be attributed to the rather small size of magnetite and GO in the hybrids [20,21]. The remnant magnetization and coercivity for thiol-functionalized MGO were 0.74 emu g^-1^ and 11.89 Oe, respectively, which were ascribed to the superparamagnetic state of magnetite nanocrystals due to the size effect. Such superparamagnetic state of the adsorbent with small remnant magnetization and coercivity at room temperature could enable the adsorbent to be readily attracted and separated by even a small external magnetic field [22]. In fact, the thiol-functionalized MGO dispersed in water solution was easily extracted from water with a magnet (Figure 3b).

**Figure 4:**
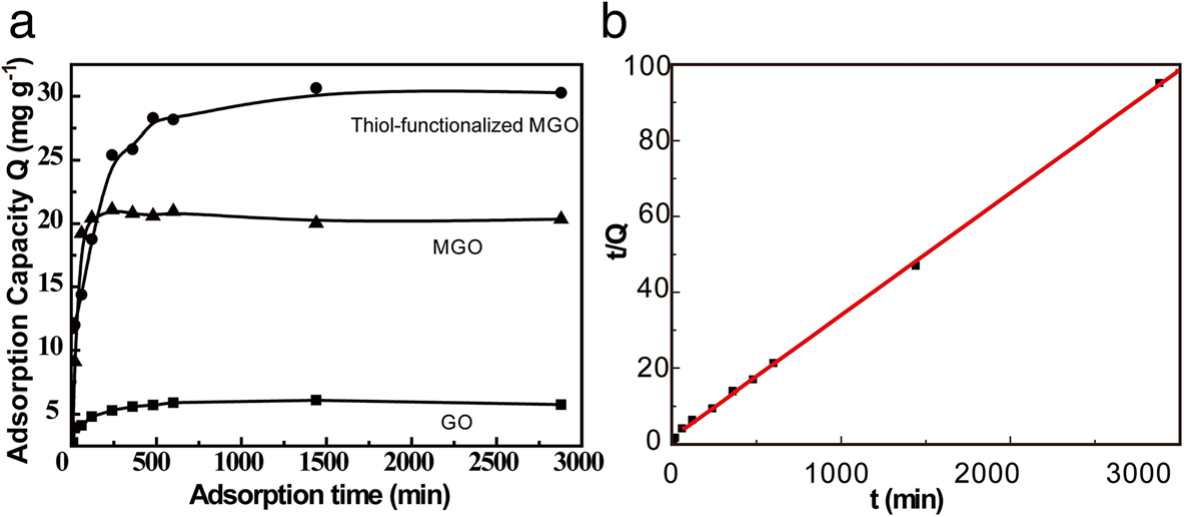
**Adsorption kinetics. (a)** Hg^2+^ adsorption kinetics of GO, MGO, and thiol-functionalized MGO, respectively. **(b)** The adsorption kinetics of thiol-functionalized MGO fits with the pseudo-second-order kinetics (initial concentration, 10 mg l^-1^).

**Figure 5:**
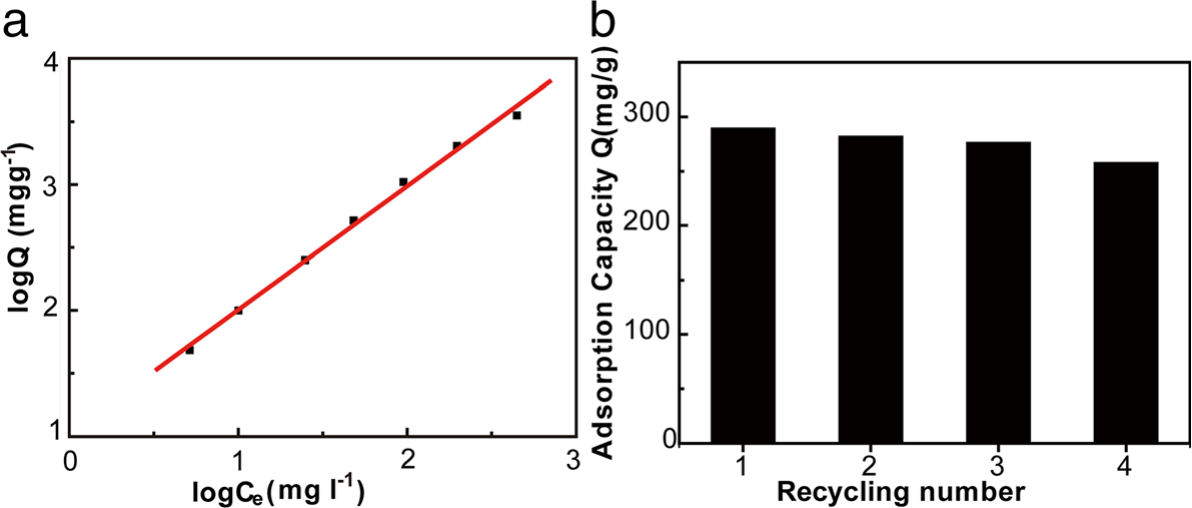
**Adsorption isotherms and adsorption capacity. (a)** Adsorption isotherms fitted with the Freundlich model (red line) for adsorption of Hg^2+^ on thiol-functionalized MGO and **(b)** adsorption capacity versus the cycling number with the initial concentration of 100 mg l^-1^ Hg^2+^.

The adsorption kinetics of Hg^2+^ by the thiol-functionalized MGO is shown Figure 4a. The initial Hg^2+^ concentration was 10 mg l^-1^. The adsorbed capacity (*Q*) of Hg^2+^ per unit mass was calculated using the following equation:

$$Q=\frac{\left(C_{0}-C_{t}\right)\times V}{W}$$

where, *Q* (mg g^-1^) is the amount of Hg^2+^ adsorbed per unit of adsorbent (mg g^-1^); *C*_0_ (mg l^-1^) and *C*_*t*_ (mg l^-1^) refer to the initial concentration of Hg^2+^ and the concentration of Hg^2+^ after the adsorption, respectively; *W* (g) is the weight of thiol-functionalized MGO; *V* (ml) is the volume of the whole solution system. After a 48-h adsorption, the solution reached a state of equilibrium. Even GO alone had a certain adsorption capacity of Hg^2+^, which was due to the formation of exchanged metal carboxylates on the surface of GO [23], while the adsorption capacity of thiol-functionalized MGO was higher than those of GO and MGO. The improved adsorption capacity of thiol-functionalized MGO could be attributed to the combined affinity of Hg^2+^ by magnetite nanocrystals and thiol groups. To determine the mechanism of Hg^2+^ adsorption from an aqueous solution by thiol-functionalized MGO, the pseudo-first-order and pseudo-second-order kinetic models were applied to interpret the adsorption data. The pseudo-second-order kinetics was presented as [24]

$$\frac{t}{Q_{t}}=\frac{1}{K_{2}Q_{e}^{2}}+\frac{t}{Q_{e}}$$

where *K*_2_ is the pseudo-second-order rate constant (g mg^-1^) and *Q*_*t*_ is the amount of Hg^2+^ adsorbed per unit of adsorbent (mg g^-1^) at time *t*. The *t*/*Q*_*t*_ versus *t* plot shown in Figure 4b indicated that the adsorption of Hg^2+^ by thiol-functionalized MGO followed the pseudo-second-order kinetic model, but not the pseudo-first-order kinetic model (Additional file 1: Figure S1a). *K*_2_ and *Q*_e_ were calculated to be 6.49E - 4 g mg^-1^ min^-1^ and 30.94 mg g^-1^, respectively. To understand how Hg^2+^ interacted with thiol-functionalized MGO, different adsorption isotherm models were used to fit the adsorption data. The data of Hg^2+^ adsorption were fit with the Freundlich isotherm model, which can be expressed as [25]

$$\log Q_{e}=\log K+\frac{1}{\;n}\log C_{e}$$

where *K* and *n* are the Freundlich adsorption isotherm constants, which are related to the relative adsorption capacity of the adsorbent and the degree of nonlinearity between solution concentration and adsorption, respectively. *K* and 1/*n* values can be calculated from the intercept and slope of the linear plot between log*C*_*e*_ and log*Q*_*e*_. Based on the plot shown in Figure 5a, *n* and *K* were calculated to be 1.02 and 10.54, respectively. However, the data did not fit the Langmuir isotherm model very well (Additional file 1: Figure S1b), indicating that the adsorption of Hg^2+^ by the adsorbent was not restricted to monolayer formations [26]. To test the reproducibility of the adsorbents, they were immersed in an aqueous solution with an initial Hg^2+^ concentration of 100 mg l^-1^ for 48 h with oscillation. The adsorption capacity for the first-time immersion was calculated to be 289.9 mg g^-1^. After being washed with diluted HCl, thiol-functionalized MGO was applied to repeat the exact same adsorption test. The obtained adsorption capacities were 282.4, 276.8, and 258.1 mg g^-1^ for the second-, third-, and fourth-time immersion, respectively, which were corresponding to 97.4%, 95.5%, and 89.0% of initial adsorption capacity. It indicated that the adsorbents could be reused.

## Conclusion

Thiol-functionalized MGO with magnetite nanoparticles was successfully synthesized using a two-step reaction. Thiol-functionalized MGO exhibited higher adsorption capacity compared to the bare graphene oxide and MGO. Its capacity reached 289.9 mg g^-1^ in the solution with an initial Hg^2+^ concentration of 100 mg l^-1^. The improved adsorption capacity could be attributed to the combined affinity of Hg^2+^ by magnetite nanocrystals and thiol groups. After being exchanged with H^+^, the adsorbent could be recycled. The adsorption of Hg^2+^ by thiol-functionalized MGO fits well with the Freundlich isotherm model and followed pseudo-second-order kinetics. The scheme reported here enables rational design of the surface properties of graphene oxide and can be used to synthesize other functionalized composites for environmental applications.

## Abbreviations

Fe(acac)3: Ferric acetylacetonate; GO: Graphene oxide; KMnO4: Potassium permanganate; MEA: 2-mercaptoethylamine; MGO: Magnetite/graphene oxide; NMP: 1-Methyl-2-pyrrolidone; SQUID: Superconducting quantum interference device; XRD: X-ray diffraction.

## Competing interests

The authors declare that they have no competing interests.

## Authors' contributions

JB and ZB designed the experiments. JB and YF performed the experiments. JB and ZB analyzed the data. JB and ZB wrote the manuscript. All authors read and approved the final manuscript.

## Supplementary Material

Additional file 1: Figure S1 (a) Adsorption kinetics fits with the pseudo-first-order model (red line) and (b) adsorption isotherm fits with the Langmuir isotherm model (red line). Click here for file
