# Supplementary material for: Thiol-functionalized magnetite/graphene oxide hybrid as a reusable adsorbent for Hg2+ removal
Source: Nanoscale Res Lett. 2013 Nov 19;8(1):486. doi: 10.1186/1556-276X-8-486 (PMC3842736; doi:10.1186/1556-276X-8-486)
Supplement: Additional file 1: Figure S1 — (a) Adsorption kinetics fits with the pseudo-first-order model (red line) and (b) adsorption isotherm fits with the Langmuir isotherm model (red line). [file 1556-276X-8-486-S1.doc]

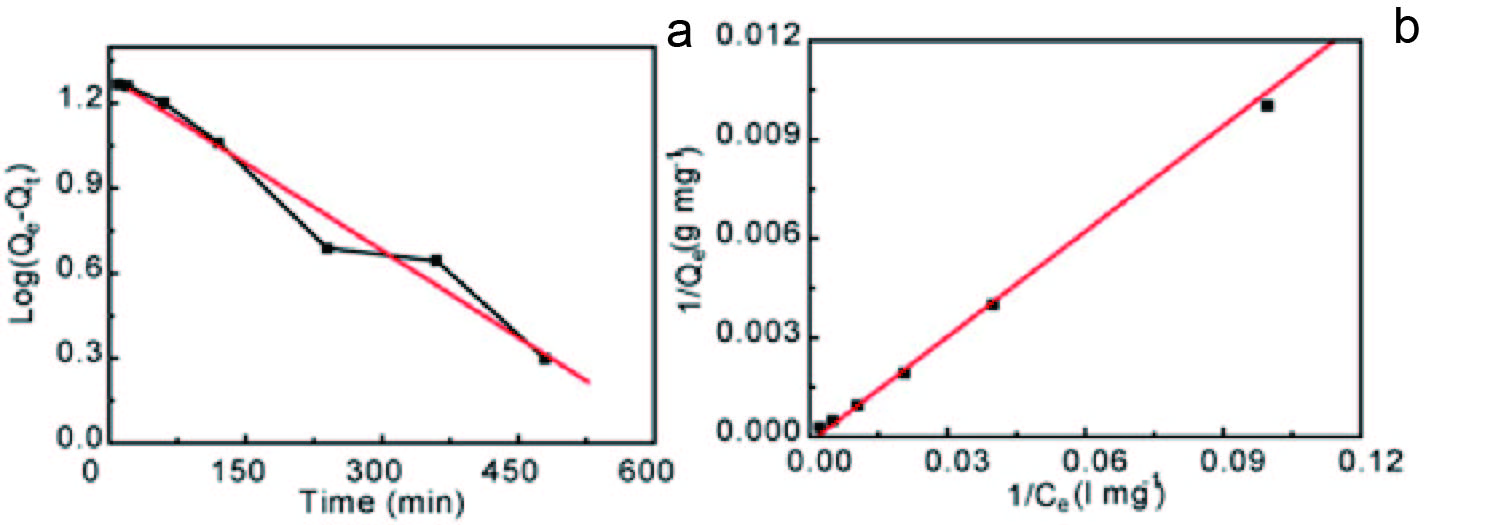


Figure S1 (a) Adsorption kinetics fits with the pseudo-first-order model (red line) and (b) adsorption isotherm fits with the Langmuir isotherm model (red line).
